# Addressing Cancer Disparities via Community Network Mobilization and Intersectoral Partnerships: A Social Network Analysis

**DOI:** 10.1371/journal.pone.0032130

**Published:** 2012-02-23

**Authors:** Shoba Ramanadhan, Carmel Salhi, Erline Achille, Nashira Baril, Kerrie D'Entremont, Milagro Grullon, Christine Judge, Sarah Oppenheimer, Chrasandra Reeves, Clara Savage, Kasisomayajula Viswanath

**Affiliations:** 1 Center for Community-Based Research, Dana-Farber Cancer Institute, Boston, Massachusetts, United States of America; 2 Department of Global Health and Population, Harvard School of Public Health, Boston, Massachusetts, United States of America; 3 New England Center of Excellence in the Elimination of Disparities, Boston Public Health Commission, Boston, Massachusetts, United States of America; 4 Mayor's Health Task Force, City of Lawrence, Lawrence, Massachusetts, United States of America; 5 Division of Public Health Practice, Harvard School of Public Health, Boston, Massachusetts, United States of America; 6 Boston Alliance for Community Health, Boston, Massachusetts, United States of America; 7 Common Pathways, Worcester, Massachusetts, United States of America; 8 Department of Society, Human Development, and Health, Harvard School of Public Health, Boston, Massachusetts, United States of America; Central Institute of Educational Technology, Canada

## Abstract

Community mobilization and collaboration among diverse partners are vital components of the effort to reduce and eliminate cancer disparities in the United States. We studied the development and impact of intersectoral connections among the members of the Massachusetts Community Network for Cancer Education, Research, and Training (MassCONECT). As one of the Community Network Program sites funded by the National Cancer Institute, this infrastructure-building initiative utilized principles of Community-based Participatory Research (CBPR) to unite community coalitions, researchers, policymakers, and other important stakeholders to address cancer disparities in three Massachusetts communities: Boston, Lawrence, and Worcester. We conducted a cross-sectional, sociometric network analysis four years after the network was formed. A total of 38 of 55 members participated in the study (69% response rate). Over four years of collaboration, the number of intersectoral connections reported by members (intersectoral out-degree) increased, as did the extent to which such connections were reported reciprocally (intersectoral reciprocity). We assessed relationships between these markers of intersectoral collaboration and three intermediate outcomes in the effort to reduce and eliminate cancer disparities: delivery of community activities, policy engagement, and grants/publications. We found a positive and statistically significant relationship between intersectoral out-degree and community activities and policy engagement (the relationship was borderline significant for grants/publications). We found a positive and statistically significant relationship between intersectoral reciprocity and community activities and grants/publications (the relationship was borderline significant for policy engagement). The study suggests that intersectoral connections may be important drivers of diverse intermediate outcomes in the effort to reduce and eliminate cancer disparities. The findings support investment in infrastructure-building and intersectoral mobilization in addressing disparities and highlight the benefits of using CBPR approaches for such work.

## Introduction

Cancer disparities (in terms of incidence, survival, and quality of life) based on social groupings, such as socioeconomic status (SES) and race/ethnicity, are a persistent problem in the United States [1], [2]. Drivers of these and other health disparities include many social determinants, such as employment and educational opportunities, access to and use of information, and environmental conditions, that have an unequal impact on population subgroups [3], [4]. When taken in the context of an ecological perspective, which recognizes individual health as a function of factors ranging from the intra- and inter-personal levels to institutional, community, and policy levels, it becomes clear that interventions are required within and across multiple levels to create sustainable change [5].

We focus here on opportunities to support multi-level action and sustainable change based in community settings, given that improved health promotion in this arena represents both an opportunity to leverage underutilized channels for health promotion as well as a vital strategy in the effort to reduce and eliminate health disparities [6], [7]. Community mobilization, such as through community coalitions and intersectoral partnerships, is a useful way to support action and social change across levels. Such efforts allow a diverse range of stakeholders and influential actors to come together and identify key health issues, plan for addressing these challenges, and then take required actions [8], [9]. Activated communities can use and build upon existing social structures and resources to engage in purposive, directed change, which may result in increased access to services, improved outreach and education efforts, or improved policies and other environmental factors [10]. Collaboration and community mobilization for health promotion have been the subjects of intense focus for government agencies and foundations in the United States over the past two decades, resulting in the formation of thousands of coalitions, alliances, and other forms of inter-organizational partnerships [11], [12]. An extensive review describing the use of collaborative partnerships for health promotion in community settings conducted by Roussos and Fawcett provides additional information [9].

As part of community mobilization efforts, intersectoral partnerships can marshal human and social capital from a wide range of partners and may be a useful solution to problems that cannot be tackled by an organization or sector in isolation [9], [12]–[14]. Diversity among partners can increase the range of resources available, not only in terms of pooling of resources or resource exchange, but synergistic creation of new and effective resources and potential to have an impact on a comprehensive set of health drivers [12], [15]. Despite the challenges of collaboration with dissimilar partners [16], such efforts have successfully been applied towards to targeting health disparities overall [17] as well as specific behaviors and diseases, such as diabetes [18], HIV/AIDS [19], and substance abuse [20].

The development of a rich and productive set of partnerships among diverse players was one of the goals driving the development of the Massachusetts Community Network for Cancer Education, Research, and Training (MassCONECT) project. This initiative was funded by the U. S. National Cancer Institute (NCI) as part of the Community Networks Program, which focused on building infrastructure in communities to reduce and eliminate cancer disparities among racial/ethnic minorities and the underserved. This program built off the success of the Special Populations Networks program, in which relationships between academics and community-based practitioners resulted in the development and delivery of culturally appropriate educational materials and capacity-building among minority investigators and practitioners, among other benefits [21]. MassCONECT brought relevant stakeholders—academics, policymakers, community leaders, and other representatives from community-based coalitions, media, and local and state government—together to reduce and eliminate cancer disparities. The program built on the foundation of four community-based coalitions to engage community leaders and policymakers in Boston, Worcester, and Lawrence, three urban communities with low-SES populations. Detailed descriptions of the project are available elsewhere [22], [23].

MassCONECT utilized a Community-based Participatory Research (CBPR) framework, which “integrates education and social action to improve health and reduce health disparities” [24]. Broadly, a CBPR approach builds on strengths and resources held by the community, combines knowledge and action to benefit of all partners, utilizes an iterative process that supports co-learning and empowerment, considers health using positive and ecological perspectives, and facilitates collaborative, equitable involvement of all partners throughout the research process [25]. This approach complements the focus on intersectoral partnerships as both perspectives place tremendous value on leveraging the knowledge and resources of diverse stakeholders in the development of practical and effective solutions to health problems [12], [25]. CBPR is also expected to deliver long-term benefits to community partners by creating capacity for advocacy and generating system changes that reduce disparities [26].

Despite the popularity of collaborative partnerships, and the growing use of CBPR for such work, the literature is rather limited in terms of the impact of network development efforts on health outcomes [9], [12]. Given that the goal of these efforts focuses on multi-level and sustainable change, impact should be measured according to those standards. Useful outcomes, then, include the following: a) relationships that develop among members of different sectors and support resource-sharing and build capacity for collective action, b) policies that are created or improved to support health, and c) systems that deliver community activities become fixtures in communities long after a particular initiative is completed [9], [13], [27], [28]. Such systems-level change is expected to drive health behavior change and ultimately have an impact on both health outcomes and health disparities [29], [30].

Given our interest in relationship development as well as the products of relationships, we turned to social network analysis to assess the development of the MassCONECT network over the first four years of the initiative. Network analysis is useful for this purpose as it allows for assessment of relationships between parties of interest, here members of collaborative networks, as well as the impacts and outcomes of these relationships [31]–[33]. This methodology allows for the study of interactions as well as the ways in which patterns of relationships drive outcomes [34]. For this project, network analysis provided an important complement to other evaluation activities, by testing the assumption that increased and improved relationships among diverse stakeholders would lead to improved cancer control and disparities reduction efforts. This analysis also allowed us to examine the potential of the network to sustain and continue the work past the funding period. Despite the potential utility of using network analysis to evaluate and intervene on community partnerships, this application is still underutilized [14]. This area of the literature is growing, as researchers assess coalitions and networks focused on general health as well as on specific health topics, such as substance abuse or cancer disparities, [13], [34]–[37].

This study adds to the field by focusing specifically on the impact of a subset of these collaborative relationships – those between members from different sectors. The purpose of the study was to explore the concepts of community mobilization and intersectoral collaboration in the context of a CBPR effort to address cancer disparities. Two research questions guided this study. First, how does participation in a CBPR infrastructure-building initiative impact the structure of the resulting network? What patterns of intersectoral relationships emerge? Second, what is the impact of intersectoral connections among network members on a diverse set of outcomes that support the reduction and elimination of cancer disparities?

## Methods

### Ethics Statement

All research procedures were approved by the Institutional Review Board at the Harvard School of Public Health and informed consent was obtained from all participants. Verbal consent was obtained through reading a consent statement that emphasized the voluntary nature of the process, the confidentiality of data, and an assurance that the participant could stop participating at any time without recourse. The ethics committee specifically approved this consent procedure and interviewers documented the consent process as part of the study protocol.

### Study Design

We conducted a cross-sectional study at the end of Year 4 of the MassCONECT initiative to describe the social network that developed over the time since the network's founding. We conducted a sociometric network analysis, meaning that we had a pre-defined network and attempted to collect data from each member about relationships to all other members on the list. This type of network analysis supports evaluation of network growth and resource exchange [33], [38]. General study results and community-specific information were presented to each of the community coalitions after the analysis was completed.

This study was conceptualized, planned, implemented, and evaluated using CBPR principles [39], by a dedicated working group which was a subset of the Community Advisory Group, which included community partners from each community as well as investigators, dissemination partners and study staff. To limit potential conflicts and biases, most working group participants ensured that colleagues would take the survey on behalf of their group; however, two working group participants answered the survey in collaboration with colleagues.

### Respondents

We defined the MassCONECT network to include 55 members who had participated in or planned events, received funding, regularly attended meetings, or supported a project/initiative directly related to MassCONECT in Years 1–4 of the initiative. This group includes the 23 original network members, who were invited to participate in the initiative by the investigators. Network members were classified as: Community-Based Organizations/Coalitions (e.g., a youth-serving agency or a coalition from one of the three communities), Researchers (either individuals or research teams), Philanthropic Organizations (e.g. foundations), Policymakers (e.g. elected and appointed officials), Providers (e.g. hospitals and health centers), and Public Sector (e.g. state and city departments of health) based on their roles at the time of entry into the MassCONECT network.

Reflecting the diversity of participants in this network, we defined “network member” as an actor that engaged with the network as an independent unit. Thus, the majority of “members” were groups, such as community coalitions or agencies. However, an individual was considered a “member” if he or she interacted with the MassCONECT network independently. For example, a junior investigator who dedicated her research time to this project without support from a larger staff and independent of her institution was considered a unique member. Similarly, independent research teams from the same university (but headed by different principal investigators) were treated as separate members given that they engaged with the network independently.

### Data Collection and Measures

Data were collected from December 2008 to February 2009 by study staff using a paper-based questionnaire. The survey was administered in-person to 26 members and by telephone to 12 members due to distance or scheduling conflicts. The vast majority of responses were given by an individual representing the given network member, but for the four coalitions, the survey was taken by a team. We administered 38 surveys, which represented 38 members and the network member was the unit of analysis. We utilized fixed list data collection methods [33], [40], meaning that we presented respondents with a roster listing all MassCONECT members. The roster was presented as a matrix with columns describing the organization (e.g. Harvard School of Public Health), the members of the team (e.g. Researcher A, Project Director B, etc.), and columns to describe interactions, if any. We presented names of organizations as well as individual members to prompt recall. This also reflected the fact that a network member could be an individual or a group based on the definition above. Respondents were asked to identify other network members they had “connected with for MassCONECT purposes” and also to identify the members they were in contact with before the MassCONECT program started in May 2005. Survey items were modeled after measures used by members of the working group in other studies [41], [42]. All survey items were finalized after cognitive interviewing, a standard technique to identify difficulties respondents face in responding to questions and ensure that questions are being understood appropriately [43].

#### Network structure

As a first step, we created network maps to describe the patterns of connections between network members. In these maps, the positions of nodes (which represent network members) in the diagrams are determined by a spring embedding algorithm, which puts network members who have many connections in the center of the diagram and also puts members who connect directly to each other or with few intermediaries closest to one another [44], [45]. Network members at the center of the diagram can be thought of as particularly involved in the network [33].

We assessed a series of network characteristics which have been shown in other settings to promote exchange of information and resources [33]. At the network-level, a measure of interest was *network density*, or the proportion of potential connections that were reported by network members. A more dense (or more highly connected) network may be useful and effective for sharing information and resources, but a more sparsely connected network may provide greater access to diverse contacts and novel resources. The point at which density transitions from being an asset to a limitation is a function of both the characteristics of the network members as well as the kind of relationship or resource transmission being studied. Regardless, a curvilinear relationship (resembling an inverse U) has been proposed between performance or spread of innovations and density [32]. We were also interested in *network centralization*, or the extent to which the network is focused around a small number of members. Networks that are highly centralized can spread information and resources effectively from the influential members, but may not be as supportive of shared decision-making and member empowerment [34] which are vital for collaborative partnerships. We also measured *network-level reciprocity*, or the proportion of connections that were reported by both members in a given pair. In other words, if Member A reports a tie to Member B and Member B also acknowledged that tie, it is considered reciprocated. Reciprocated connections tend to be stronger and are better supports of exchanges than connections that are only reported by one half of a pair. High reciprocity can indicate stronger relationships, but can also indicate greater clustering of groups within the network, which may limit exposure to/spread of new ideas [32].

A useful descriptive measure (assessed per network member) is *degree*, or the number of connections a given member has in the network. We then focused on *out-degree*, or the number of connections a given member reported to other members. This measure represents the set of connections that may be functionally useful to respondents [46]; here, potential collaborators with whom a member may engage. We also calculated *betweenness*, or the frequency with which a member serves as the most efficient way for other members to connect. Members with high betweenness occupy a strategic position in the network as they can link (or not link) other members, regardless of the overall number of connections they possess [32].

#### Intersectoral connections

We then narrowed the analysis to focus on intersectoral connections in the network. Analysis of *intersectoral network density* summarizes the percentage of potential connections that were realized both within each group (e.g. among Providers), but also between groups (e.g. Providers' connections with Researchers). This metric counts all ties between members of a group, so a tie between a Researcher and a Provider is counted regardless of whether one or both parties noted that connection in the survey. We used the UCINET density-by-groups procedure for this analysis [44]. For individual-level analyses, we focused on *intersectoral out-degree*, which represents the number of connections a given member reported to network members belonging to other sectors. This measure combines our interest in intersectoral connections with the focus on connections that may be perceived by the respondent as functionally useful. We used UCINET matrix manipulation routines to calculate this metric for each individual. Last, we assessed *intersectoral reciprocity*, which assesses the percentage of reported ties that are reciprocal. For the group-level comparisons, the measure assesses the percentage of ties that are part of reciprocated connections and then summarizes these patterns by group, here by sector [44], [45]. As opposed to intersectoral network density, the statistic is calculated for each group regarding all of the other groups. Thus, the percentage of reciprocated connections reported by Researchers regarding Providers may not be the same as the percentage of reciprocated connections reported by Providers regarding Researchers. At the individual-level, we calculated the percentage of reported intersectoral ties that were reciprocated. We used the UCINET reciprocity procedure for these measures [44].

#### Key outcomes

To assess the impact of community mobilization and intersectoral collaboration, we focused on three goals of the MassCONECT effort to reduce and eliminate cancer disparities: community activities, grants and publications, and policy engagement. Again, this draws on the social ecological model and CBPR theories to recognize the importance of relationships between organizations/in communities, as well as the need for multi-level and multi-pronged health promotion efforts to drive changes in health behavior and health outcomes [29], [47]. We created an index to measure activity in each of these categories and respondents were asked to focus on MassCONECT-related work for each set of items. The four-item community activities index summarized reports that the member participated in, planned, and/or presented any of the following: activities hosted in the community, events to increase access to cancer-related services, events to improve the ability of community-based organizations to work with the media, or technical assistance. Community outreach and supports for improved prevention services were in line with the network goal to increase cancer control programming in underserved areas and build capacity for this work at the community level. The three-item grants and publications index summarized reports of submitting or receiving an award for a CBPR grant as well as participation in the development of a peer-reviewed manuscript. This outcome reflects the goal of increasing capacity among network members to engage in CBPR research in order to increase use of evidence-based cancer control interventions and decrease cancer disparities. The two-item policy engagement index summarized reports of engagement in policy development/implementation and engagement with state or local policymakers. Policy change is one of the markers of sustainable, system-focused change for communities.

### Data Analysis

Network analysis requires dedicated software to assess relational data, and we used UCINET-6 [44]. This software package includes procedures developed specifically for network data, which contain observations that are not independent and do not meet the assumptions of classical statistical techniques. Significance tests presented in this analysis are based on random permutations of matrices as is appropriate for relational data. Here, the significance levels were determined based on distributions created from 10,000 random permutations. We used linear regression procedures developed for network data for hypothesis testing [45], [48]. Descriptive measures were calculated using standard UCINET procedures developed for network data. For the first set of regression analyses, the multiple linear regression models included our predictor of interest, intersectoral out-degree, three collaboration outcomes, and several theoretically important covariates: City, Original vs. New Member, and Member Sector (e.g. CBO/Coalition). Dummy variables were created for the City and Sector variables. We tested the addition of other potential covariates, but did not find additional significant contributors to the model. The same process was used for the second set of analyses, in which the multiple linear regression models included our second predictor of interest, intersectoral reciprocity, three collaboration outcomes, and the same set of covariates. Again, the addition of other potential covariates did not improve the model and thus the model was left in this form. We could not analyze our two predictors of interest simultaneously because they were too highly correlated with each other. We were also unable to include intrasectoral out-degree and intrasectoral reciprocity in the models for the same reason [49].

## Results

A total of 38 of 55 network members completed the survey (69% response rate). Respondents included 11 Community-Based Organizations/Coalitions, 1 Philanthropic Organization, 2 Policymakers, 4 Providers, 6 Public Sector Agencies, and 14 Research Members. Additional details are provided in Table 1.

**Table 1:** Descriptive Characteristics of 38 Members Participating in the MassCONECT Network Analysis at Year 4.

	Frequency	Percent (%)
*Descriptive Characteristics*		
Network Members, by Sector		
CBO/Coalition	11	29
Researcher	14	37
Philanthropic	1	3
Policymaker	2	5
Provider	4	11
Public	6	16
City of Origin		
Boston	26	68
Lawrence	5	13
Worcester	7	18
Membership Tenure		
Original Network Member	23	61
*Key Network Outcomes*		
Community Activities Index (4 items)	Mean: 1.97	SD: 1.42
Publications and Grants Index (3 items)	Mean: 2.29	SD: 1.01
Policy Engagement Index (2 items)	Mean: 1.11	SD: 1.29

### Network Structure

**Figure 1:**
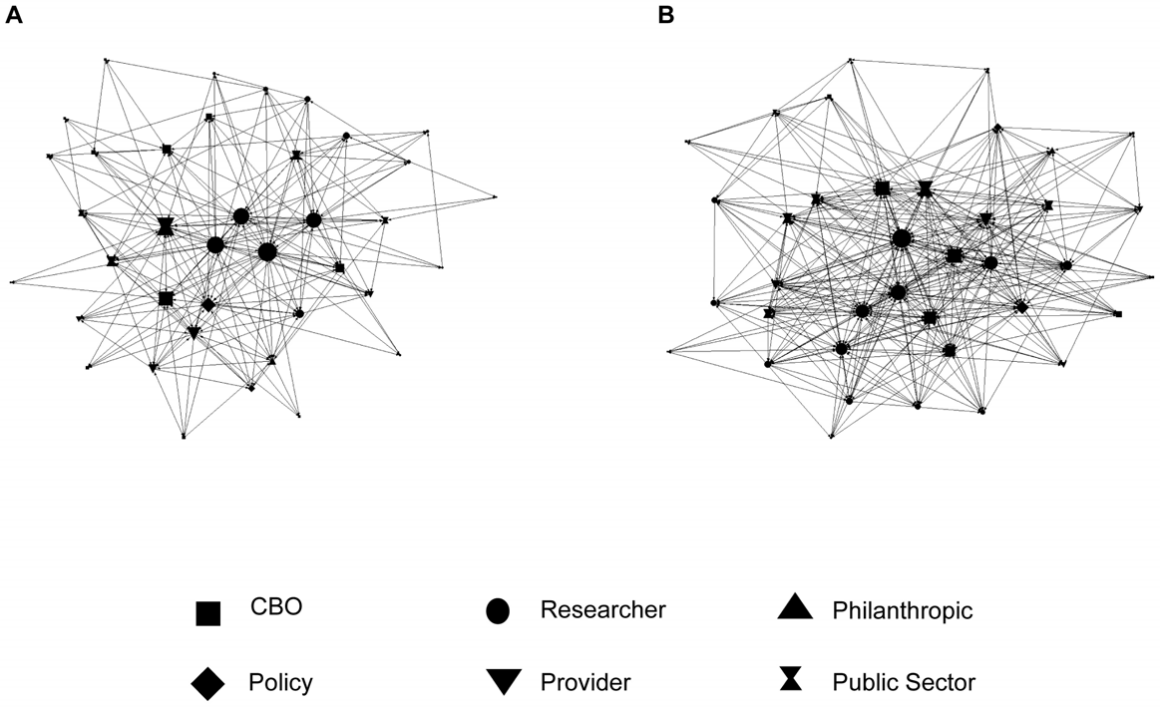
Connections among 38 MassCONECT members at network inception (panel A) and Year 4 (panel B). Lines represent connections between network members, arrows reference direction(s) of connections. Node size represents degree, or number of connections per member.

The network diagrams presented in Figure 1 provide a visual aid to understand the changes in the network from Inception to Year 4. First, there is an increase in the number of connections (represented by lines between shapes, which represent members) from Network Inception to Year 4. The network density, or the proportion of all possible ties reported, was 16% at Network Inception and 35% at Year 4. The figure also highlights increasing diversity of key players. Compared to Network Inception, the map at Year 4 has a large number of network members who appear to be important to the network and they come from a wider range of sectors. This interpretation is supported by examining the network centralization, or the extent to which the network is focused on a small number of members, which decreased from 61% to 44% when considering out-degree (or outgoing connections). A final important network-level metric is reciprocity. We found that reciprocity (or connections that are reported by both members of a pair) was 19% at Network Inception and 54% at Year 4.

Averages across the network for a series of member-level attributes also provide a useful picture. The average degree, or number of connections for a network member, was 10.21 (SD = 6.28) at Network Inception and 16.58 (SD = 7.97) at Year 4. The average out-degree, or number of connections reported by each network member, increased from 6.08 (SD = 6.31) at Network Inception to 12.76 (SD = 8.81) at Year 4. We also measured betweenness, which decreased from 36.37 at Network Inception to 25.90 at Year 4.

### Intersectoral Connections: Sector-level Patterns

To examine intersectoral connections at a higher level, we assessed intersectoral density, or the density of connections between members of different sectors. As seen in Table 2, we found increases in the density of connections within and between most sectors between Network Inception and Year 4. Community-Based Organizations and Coalitions reported increases in connections with all other groups, such as a 16% increase in connections with researchers (from 19% to 35%). Similarly, Research members reported increased density of connections with all groups. Results for Philanthropic and Policymaker members are harder to interpret given that they had only 1 and 2 members, respectively. Public Sector Agencies reported increases in connections with all other groups, with the exception of Philanthropic Organizations (n = 1). We also found that a number of groups demonstrated increases in the density of within-group connections. We extended our interest in connection patterns to focus on intersectoral reciprocity at the sector level. As seen in Table 3, we found increased reciprocity of connections within and between most sectors, with an average change of 29% increase. The major exceptions to this pattern related to the Philanthropic Sector, but this is likely a function of having only one respondent in this category.

**Table 2:** Change in Density of Connections (Percentages) Within and Between Sectors from Inception to Year 4, n = 38.

	CBO/Coalition (n = 11)	Researcher (n = 14)	Philanthropic (n = 1)	Policymaker (n = 2)	Provider (n = 4)	Public Sector (n = 6)
CBO/Coalition	20 (16–36)	16 (19–35)	9 (27–36)	9 (32–41)	7 (18–25)	26 (24–50)
Researcher		25 (41–66)	7 (29–36)	18 (28–46)	16 (27–43)	14 (30–44)
Philanthropic			n/a	50 (0–50)	0 (50–50)	0 (17–17)
Policymaker				0 (100–100)	13 (62–75)	17 (41–58)
Provider					33 (50–83)	17 (33–50)
Public Sector						13 (54–67)

Density levels (percentages) at Network Inception and Year 4 provided in parentheses.

**Table 3:** Change in Average Reciprocity of Connections (Percentages) Within and Between Sectors from Inception to Year 4, n = 38.

	CBO/Coalition (n = 11)	Researcher (n = 14)	Philanthropic (n = 1)	Policymaker (n = 2)	Provider (n = 4)	Public Sector (n = 6)
CBO/Coalition	22 (33–55)	29 (60–89)	25 (0–25)	33 (67–100)	11 (60–71)	16 (60–76)
Researcher	55 (11–66)	48 (19–67)	−5 (25–20)	20 (20–40)	31 (8–39)	53 (20–73)
Philanthropic	n/a	0 (100–100)	0 (0–0)	100 (0–100)	0 (0–50)	0 (0–0)
Policymaker	22 (34–56)	32 (25–57)	100 (0–100)	0 (0–0)	67 (0–67)	58 (25–83)
Provider	6 (50–56)	65 (25–90)	0 (100–100)	100 (0–100)	27 (33–60)	−24 (80–56)
Public Sector	36 (21–57)	31 (45–76)	0 (0–0)	33 (50–83)	5 (58–63)	15 (25–40)

Reciprocity (percentages) at Network Inception and Year 4 provided in parentheses.

Column indicates the group affiliation of the member reporting the connection and row indicates the group affiliation of the member about whom the connection is reported.

### Intersectoral Connections: Member-level Patterns

We found that the average intersectoral out-degree for members was 4.21 connections (SD = 4.88) at Network Inception compared to 8.71 connections (SD = 6.52) at Year 4. For intersectoral reciprocity, we found that the percentage of reported connections that were reciprocated increased from 0.16 (SD = 0.13) at Network Inception to 0.49 (SD = 0.18) at Year 4.

### Key Network Outcomes

The three outcome indices measured the extent to which members engaged in stated network goals. The mean response for the community activities index was 1.97 out of 4 (SD = 1.42); the mean for the publications and grants index was 2.29 out of 3 (SD = 1.01); and the mean for the policy engagement index was 1.11 out of 2 (SD = 1.29).

### Impact of Intersectoral Collaboration on Key Outcomes

We examined the impact of intersectoral out-degree (the number of intersectoral connections reported by a given member) on three key network outcomes. Results are presented in Table 4. We found a positive and statistically significant relationship between intersectoral out-degree and the community activities index (β = 0.15, p = 0.002), controlling for important covariates, including the location of the network member, whether or not the member was part of the original network, and sector affiliation. Similarly, we found a positive and borderline significant relationship between intersectoral out-degree and the grants and publications index, controlling for the same covariates (β = 0.09, p = 0.06). We found a positive and statistically significant relationship between intersectoral out-degree and the policy engagement index (β = 0.05, p = 0.05).

**Table 4:** Association between Three Collaboration Outcomes and Intersectoral Degree (Model 1) and Intersectoral Reciprocity (Model 2) at Year 4, controlling for important covariates^1^ (n = 38).

	Outcome 1: Community Activities Index	Outcome 2: Grants and Publications Index	Outcome 3: Policy Engagement Index
**Model 1**			
Intercept	0.96	−0.10	0.85
Intersectoral Out-degree	0.15**	0.09+	0.05*
R^2^	0.64	0.53	0.40
**Model 2**			
Intercept	0.75	−0.65	0.49
Intersectoral Reciprocity	3.59**	3.46**	1.35+
R^2^	0.57	0.67	0.38

Key: + p-value less than or equal to 0.10,

*less than or equal to 0.05,

**less than or equal to 0.01.

1 : Models control for city affiliation, member status (original vs. new member), and member sector.

We then assessed the impact of intersectoral reciprocity on our three outcomes of interest, as seen in Table 4. We found a positive and statistically significant relationship between intersectoral reciprocity and the community activities index (β = 3.59, p = 0.004), controlling for important covariates, including the location of the network member, whether or not the member was part of the original network, and sector. We found a positive and statistically significant relationship between intersectoral reciprocity and the grants and publications index (β = 3.46, p = 0.003), controlling for the same covariates. We found a positive and borderline significant relationship between intersectoral reciprocity and policy engagement, controlling for the same covariates (β = 1.35, p = 0.07).

## Discussion

This study describes a successful community mobilization effort that resulted in increased intersectoral partnerships and generated important short-term collaboration outcomes in the first four years of development. A diverse set of partners were engaged in a CBPR effort to reduce and eliminate cancer disparities, with purposive and directed effort in the areas of community activities, grants and publications, and policy engagement. Successful network development efforts such as those described here point to the utility of investments in infrastructure building, as well as the promise of using CBPR approaches for such endeavors.

The first hallmark of successful infrastructure building can be seen in the network structure that developed over the first four years of the MassCONECT project. Overall, we found increased connectedness and reciprocity of connections, which prime the network to support resource exchange and collaboration [32], [45]. A similar pattern was found for connections between most sectors, which is expected to correlate with access to an increasingly diverse range of resources for network members [15]. The ability for an external initiative to support infrastructure building in intersectoral networks has been demonstrated elsewhere in health promotion settings [13], [36]. These patterns reflect the spirit of community mobilization, which requires opportunities for diverse stakeholders to engage in the planning and production of change [11]. Certainly, both increased connectivity and collaboration with dissimilar partners come at a cost to network members and there is likely a threshold beyond which the cost of maintaining an extensive and diverse set of relationships exceeds the marginal utility of those relationships.

In addition to infrastructure development, we also found that intersectoral connections supported key intermediate outcomes for addressing cancer disparities: community engagement, grants and publications, and policy engagement. These findings are consistent with other research suggesting that the number of diverse connections and the strength of connections are important drivers of impact for collaborative efforts [11], [46], [50]. The volume of partnership outputs demonstrates the impact of network development. In the first four years of the network's existence, the group delivered 117 community activities (of which 51 were focused on cancer), which reached over 13,000 individuals; developed 26 outreach materials, over 17,000 copies of which were distributed; generated 23 publications; and successfully applied for 7 leveraged grants [51]. The seven grants that were developed out of this work include new CBPR projects, offering opportunities for network members to continue to collaborate, build capacity in the communities, and create long-term, sustainable change. Findings of network growth, increased collaboration among diverse types of partners, and support for goals tied to reducing and eliminating cancer disparities are consistent with the results of network analysis studies from other CNPs, such as the Tampa Bay Community Cancer Network [37], [52], and the WINCART program from California [36]. These studies point to the potential gains from collaboration among diverse partners, development of trust, and the impacts of capacity-building and CBPR to address disparities. At the same time, they raise a series of important challenges, including difficulties sustaining networks in a time of severe resource constraints.

By creating changes across multiple levels and in multiple sectors, the initiative created sustainable environmental changes, which are necessary to impact health, and in this case cancer disparities [9]. Such collaboration allows for creation of new assets, exchange and development of knowledge, ability to leverage complementary skills and resources, and improved efficiency of interaction between partners [53]. Also, from a capacity-building perspective, increased collaboration between organizations provides an opportunity for skill-sharing and skill transfer, so that the overall capacity of the network increases [54]. Increased capacity in this context may also improve opportunities to reduce the research-to-practice gap and bring evidence-based interventions to underserved communities in an appropriate manner [30]. These benefits point to the utility of investing in networks and allowing for the necessary time and resource commitment that form the basis for future collaboration and benefit.

In this network, a series of factors may have had a particular impact on the success of building intersectoral connections and engaging members in collaborative work. First, infrastructure and partnership development as well as resource exchange among partners were among the major goals of the MassCONECT project. The program was explicitly designed to leverage existing social structures and resources within influential community coalitions and build partnerships among diverse types of members. Making network development an explicit goal and communicating this over the life of the grant provides a focus both on short-term collaboration, but more importantly, on long-term relationship development. Another supporting feature was that network members had a common and compelling goal, to reduce and eliminate health disparities. Such a focus is thought to help build consensus and motivate action among diverse network members [9]. Additionally, the application of CBPR approaches and frameworks emphasized collaboration among, participation from, and benefit to all partners, which all support effective network development, as found in a comparable network [36]. The social network analysis presented here is an excellent example of the diverse benefits of intersectoral collaboration. The impetus for the network analysis came from community partners, the study was executed by a team of researchers and community partners, and the work resulted not only in the sharing of results to each community, but also in the development of a one-day workshop on networks for staff of community-based organizations in the three communities. Tools developed for that workshop are still being shared and utilized through one of the leveraged grants, including a planning tool for strategic assessment and utilization of partnerships.

There are some limitations that help place the results in context. First, the results may be influenced by differences between respondents and non-respondents. Although we had a high response rate, some network members did not complete the survey. These members may have been less engaged with their counterparts than respondents and thus we may have studied the members who took most advantage of the network. Second, the majority of respondents received financial or other resources through participation in the MassCONECT network and their desire to demonstrate success may have influenced their responses. Third, the data are cross-sectional; thus causation cannot be assessed. However, a connection must exist between individuals at the time of or before collaboration, so the directionality assumed seems plausible. Fourth, there was some heterogeneity in the data collection methods, but adjustment for survey mode did not impact the findings. Last, we studied a single network and therefore cannot generalize these findings to other networks or situations. Despite these limitations, the study provides a useful case study, assessing community mobilization and intersectoral collaboration with a systems perspective, focused on intermediate outcomes that impact sustainable change in the area of cancer disparities.

In summary, this study suggests that infrastructure-building efforts to support community mobilization and intersectoral collaboration can prime local systems to create sustainable change and reduce and eliminate cancer disparities. The challenge will be in maintaining and continuing to invest in these networks, so that the networks remain dynamic and can adapt to meet new challenges and offer new benefits to partners [14], [55]. Future studies that include longitudinal data can provide deeper understanding of the mechanisms by which intersectoral partnerships and community mobilization can lead to effective efforts to tackle health problems. Similarly, detailed characterization of relationships (both in terms of structure as well as content exchanged) will allow for proactive network development to support collaborative efforts. Given the high cost of developing and maintaining connections in a network, particularly with diverse partners, strategic network development is of the essence, with a consistent focus on important benefits to all partners as well as the ultimate goal of reducing and eliminating health disparities.

## References

[1] Freeman H (1991). Race, poverty, and cancer.. Journal of the National Cancer Institute.

[2] Trans-HHS Cancer Health Disparities Progress Review Group (2004). Making Cancer Health Disparities History.

[3] Viswanath K, Thomson GE, Mitchell F, WIlliams MB (2006). Public communication and its role in reducing and eliminating health disparities.. Examining the health disparities research plan of the National Institutes of Health: Unfinished business.

[4] Williams DR, Costa MV, Odunlami AO, Mohammed SA (2008). Moving upstream: how interventions that address the social determinants of health can improve health and reduce disparities. .. Journal of Public Health Management and Practice.

[5] McLeroy KR, Bibeau D, Steckler AB, Glanz K (1988). An Ecological Perspective on Health Promotion Programs.. Health Education Quarterly.

[6] Griffith DM, Allen JO, DeLoney EH, Robinson K, Lewis EY (2010). Community-based organizational capacity building as a strategy to reduce racial health disparities.. Journal of Primary Prevention.

[7] Maibach EW, Van Duyn MAS, Bloodgood B (2006). A marketing perspective on disseminating evidence-based approaches to disease prevention and health promotion.. Preventing Chronic Disease.

[8] Minkler M, Wallerstein N, Wilson N, Glanz K, Rimer B, Viswanath K (2008). Improving health through community organization and community building. .. Health behavior and health education: Theory, research and practice. 4th ed.

[9] Roussos ST, Fawcett SB (2000). A Review of Collaborative Partnerships as a Strategy for Improving Community Health.. Annual Reviews of Public Health.

[10] Bracht N, Tsouros A (1990). Principles and strategies of effective community participation.. Health Promotion International.

[11] Butterfoss FD, Goodman RM, Wandersman A (1996). Community coalitions for prevention and health promotion: Factors predicting satisfaction, participation and planning. .. Health Education Quarterly.

[12] Lasker RD, Weiss ES, Miller R (2001). Partnership Synergy: A Practical Framework for Studying and Strengthening the Collaborative Advantage.. The Milbank Quarterly.

[13] Provan KG, Nakama L, Veazie MA, Teufel-Shone NI, Huddleston C (2003). Building community capacity around chronic disease services through a collaborative interorganizational network. .. Health Education and Behavior.

[14] Provan KG, Veazie MA, Staten LK, Teufel-Shone NI (2005). The Use of Network Analysis to Strengthen Community Partnerships.. Public Administration Review.

[15] Burt RS (1992). Structural Holes: The Social Structure of Competition.

[16] Goerzen A, Beamish PW (2005). The Effect of Alliance Network Diversity on Multinational Enterprise Performance.. Strategic Management Journal.

[17] Hennessey LS, Smith ML, Esparza AA, Hrushow A, Moore M (2005). The community action model: a community-driven model designed to address disparities in health.. American Journal of Public Health.

[18] Giachello AL, Arrom JO, Davis M, Sayad JV, Ramirez D (2003). Reducing diabetes health disparities through community-based participatory action research: the Chicago Southeast Diabetes Community Action Coalition.. Public Health Reports.

[19] Parker R, Aggleton P (2003). HIV and AIDS-related stigma and discrimination: a conceptual framework and implications for action.. Social Science & Medicine.

[20] Shults RA, Elder RW, Nichols JL, Sleet DA, Compton R (2009). Effectiveness of multicomponent programs with community mobilization for reducing alcohol-impaired driving.. American Journal of Preventive Medicine.

[21] Van Duyn MA, Reuben SH, Macario E (2006). Special populations networks: Themes and lessons learned.. Cancer.

[22] Koh HK, Oppenheimer SC, Massin-Short SB, Emmons KM, Geller AC (2010). Translating Research Evidence Into Practice to Reduce Health Disparities: A Social Determinants Approach.. American Journal of Public Health.

[23] Emmons KM, Viswanath K, Colditz GA (2008). The Role of Transdisciplinary Collaboration in Translating and Disseminating Health Research: Lessons Learned and Exemplars of Success.. American Journal of Preventive Medicine.

[24] Wallerstein NB, Duran B (2006). Using community-based participatory research to address health disparities.. Health Promotion Practice.

[25] Israel BA, Schulz AJ, Parker EA, Becker AB (1998). Review of community-based research: Assessing partnership approaches to improve public health. .. Annu Rev Public Health.

[26] Viswanathan M, Ammerman A, Eng E, Gartlehner G, Lohr KN (2004). Community-Based Participatory Research: Assessing the Evidence. Report/Technology Assessment No. 99..

[27] Koelena MA, Vaandragerb L, Colomérc C (2001). Health promotion research: dilemmas and challenges.. Journal of Epidemiology and Community Health.

[28] Varda D, Shoup JA, Miller S (2011). A systematic review of collaboration and network research in the public affairs literature: Implications for public health practice and research.. American Journal of Public Health.

[29] Simons-Morton B, Mcleroy KR, Wendel ML (2011). Behavior Theory in Health Promotion Practice and Research.

[30] Wallerstein N, Duran B (2010). Community-based Participatory Research Contributions to Intervention Research: The Intersection of Science and Practice to Improve Health Equity.. American Journal of Public Health.

[31] Scott J (2000). Social Network Analysis: A Handbook.

[32] Valente TW (2010). Social Networks: Models, Methods, and Applications.

[33] Wasserman S, Faust K (1994). Social Network Analysis: Methods and Analysis.

[34] Valente TW, Coronges KA, Stevens GD, Cousineau MR (2008). Collaboration and competition in a children's health initiative coalition: A network analysis.. Evaluation and Program Planning.

[35] Valente TW, Chou CP, Pentz MA (2007). Community coalitions as a system: effects of network change on adoption of evidence-based substance abuse prevention.. American Journal of Public Health.

[36] Valente TW, Fujimoto K, Palmer P, Tanjasri SP (2010). A Network Assessment of Community-Based Participatory Research: Linking Communities and Universities to Reduce Cancer Disparities.. American Journal of Public Health.

[37] Luque JS, Tyson DM, Lee J, Gwede CK, Vadaparampil ST (2010). Using social network analysis to evaluate community capacity building of a regional community cancer network.. Journal of Community Psychology.

[38] Valente TW, Carrington PJ, Scott J, Wasserman S (2005). Network Models and Methods for Studying the Diffusion of Innovations.. Models and Methods in Social Network Analysis.

[39] Zenk SN, Schulz AJ, House JS, Benjamin A, Kannan S, Israel BA, Eng E, Schulz AJ, Parker EA (2005). Application of CBPR in the Design of an Observational Tool.. Methods in Community Based Participatory Research.

[40] Luke DA, Harris JK (2007). Network analysis in public health: history, methods, and applications.. Annu Rev Public Health.

[41] Ramanadhan S, Wiecha JL, Gortmaker SL, Emmons KM, Viswanath K (2010). Informal Training in Staff Networks to Support Dissemination of Health Promotion Programs.. American Journal of Health Promotion.

[42] Ramanadhan S, Kebede S, Mantopoulos J, Bradley EH (2010). Network-based social capital and capacity-building programs: An example from Ethiopia.. Human Resources for Health.

[43] Tourangeau R, Rips LJ, Rasinski K (2000). The psychology of survey response.

[44] Borgatti SP, Everett MG, Freeman LC (2005). UCINET for windows: Software for social network analysis.

[45] Hanneman RA, Riddle M (2005). Introduction to social network methods.

[46] Hansen MT (1999). The search-transfer problem: The role of weak ties in sharing knowledge across organization subunits.. Administrative Science Quarterly.

[47] Sandoval JA, Lucero J, Oetzel J, Avila M, Belone L (2011). Process and outcome constructs for evaluating community-based participatory research projects: a matrix of existing measures..

[48] Krackhardt D (1988). Predicting with social networks: Nonparametric multiple regression analysis of dyadic data.. Social Networks.

[49] Nunnally JC, Bernstein IH (1994). Psychometric Theory.

[50] Powell WW, Koput KW, Smith-Doerr L, Owen-Smith J, Andrews SB, Knoke D (1999). Network Position and Firm Performance: Organizational Returns to Collaboration in the Biotechnology Industry.. Networks In and Around Organizations, Volume 16 (Research in the Sociology of Organizations).

[51] Koh H (2009). MassCONECT Year 4 Report, Submitted to the National Cancer Institute.

[52] Luque JS, Tyson DM, Bynum SA, Noel-Thomas S, Wells KJ (2011). A social network analysis approach to understand changes in a cancer disparities community partnership network.. Annals of Anthropological Practice.

[53] Dyer JH, Singh H (1998). The Relational View: Cooperative Strategy and Sources of Interorganizational Competitive Advantage.. Academy of Management Review.

[54] Powell WW, Koput KW, Smith-Doerr L (1996). Interorganizational Collaboration and the Locus of Innovation: Networks of Learning in Biotechnology.. Administrative Science Quarterly.

[55] Kanter RM (1994). Collaborative Advantage: The Art of Alliances.. Harvard Business Review.

